# Jak2 Inhibitor AG490 Improved Poststroke Central and Peripheral Inflammation and Metabolic Abnormalities in a Rat Model of Ischemic Stroke

**DOI:** 10.3390/antiox10121958

**Published:** 2021-12-07

**Authors:** Ya-Yu Wang, Shih-Yi Lin, Cheng-Yi Chang, Chih-Cheng Wu, Wen-Ying Chen, Su-Lan Liao, Yu-Fan Chen, Wen-Yi Wang, Chun-Jung Chen

**Affiliations:** 1 Department of Family Medicine, Taichung Veterans General Hospital, Taichung City 407, Taiwan; yywang@vghtc.gov.tw; 2 Institute of Clinical Medicine, National Yang-Ming Chiao Tung University, Taipei City 112, Taiwan; sylin@vghtc.gov.tw; 3 Center for Geriatrics and Gerontology, Taichung Veterans General Hospital, Taichung City 407, Taiwan; 4 Department of Surgery, Feng Yuan Hospital, Taichung City 420, Taiwan; c.y.chang.ns@gmail.com; 5 Department of Anesthesiology, Taichung Veterans General Hospital, Taichung City 407, Taiwan; chihcheng.wu@gmail.com; 6 Department of Financial Engineering, Providence University, Taichung City 433, Taiwan; 7 Department of Data Science and Big Data Analytics, Providence University, Taichung City 433, Taiwan; 8 Department of Veterinary Medicine, National Chung Hsing University, Taichung City 402, Taiwan; wychen@dragon.nchu.edu.tw; 9 Department of Medical Research, Taichung Veterans General Hospital, Taichung City 407, Taiwan; slliao@vghtc.gov.tw; 10 Department of Medical Laboratory Science, I-Shou University, Kaohsiung City 840, Taiwan; chentina830@gmail.com; 11 Department of Nursing, Hung Kuang University, Taichung City 433, Taiwan; walice@sunrise.hk.edu.tw; 12 Department of Medical Laboratory Science and Biotechnology, China Medical University, Taichung City 404, Taiwan

**Keywords:** hyperglycemia, IL-6, insulin resistance, neuroinflammation, stroke

## Abstract

Poststroke hyperglycemia and inflammation have been implicated in the pathogenesis of stroke. Janus Kinase 2 (Jak2), a catalytic signaling component for cytokine receptors such as Interleukin-6 (IL-6), has inflammatory and metabolic properties. This study aimed to investigate the roles of Jak2 in poststroke inflammation and metabolic abnormality in a rat model of permanent cerebral ischemia. Pretreatment with Jak2 inhibitor AG490 ameliorated neurological deficit, brain infarction, edema, oxidative stress, inflammation, caspase-3 activation, and Zonula Occludens-1 (ZO-1) reduction. Moreover, in injured cortical tissues, Tumor Necrosis Factor-α, IL-1β, and IL-6 levels were reduced with concurrent decreased NF-κB p65 phosphorylation, Signal Transducers and Activators of Transcription 3 phosphorylation, Ubiquitin Protein Ligase E3 Component N-Recognin 1 expression, and Matrix Metalloproteinase activity. In the in vitro study on bEnd.3 endothelial cells, AG490 diminished IL-6-induced endothelial barrier disruption by decreasing ZO-1 decline. Metabolically, administration of AG490 lowered fasting glucose, with improvements in glucose intolerance, plasma-free fatty acids, and plasma C Reactive Proteins. In conclusion, AG490 improved the inflammation and oxidative stress of neuronal, hepatic, and muscle tissues of stroke rats as well as impairing insulin signaling in the liver and skeletal muscles. Therefore, Jak2 blockades may have benefits for combating poststroke central and peripheral inflammation, and metabolic abnormalities.

## 1. Introduction

Cerebral vascular stenosis, occlusion, or rupture can cause insufficient blood flow and perfusion in the brain, potentially leading to hypoxia, neurological deficits, physical disability, and even death [1]. In a recently published review, it is reported that in patients with acute ischemic stroke, early intravenous thrombolysis improves the likelihood of minimal or no disability by around one third within 3–4.5 h of presentation [2]. Besides this, acute stroke patients due to large-vessel occlusions are more likely to be functionally independent when treated with mechanical thrombectomy within 6 h of presentation. However, in spite of these scientific advances for acute stroke treatment evolving rapidly, either from clinical trials and the everyday experiences from different centers, only up to 10% of ischemic stroke patients actually receive these therapies due to constraints such as narrow therapeutic windows, and many patients progress into persistent disability [3,4,5,6]. Therefore, there still is a huge unmet medical need for newer therapies for stroke.

Acute stroke is accompanied by the production of Reactive Oxygen Species (ROS), inflammation, and transient hyperglycemia [7,8], all of which contributed to neuronal injury, and correlated with outcomes in patients with stroke [9,10]. Notably, after the permanent interruption of blood supply, the ischemic penumbra, a region vulnerable to injuries, can still be salvageable. It has been proposed that an early intervention with those deleterious responses following ischemic stroke may help to modulate the second wave of brain injury. Experimental studies have shown that interventions involving ROS, inflammation, and glucose metabolism diminish stroke-induced neuronal injuries in rodent models [7,8,11].

Interleukin-6 (IL-6) is a pleiotropic cytokine, adipokine, and myokine [12]. Accumulating evidence indicates that IL-6 is involved in oxidative stress and inflammation in adipose, skeletal muscles, and hepatic tissues [13,14,15,16] in association with insulin resistance and impaired fasting glucose [17]. Receptors involved in the recognition of IL-6 include the ligand-specific IL-6 receptor (IL-6R) and the signal-transducing receptor gp130 [12]. Once engagement of ligands and receptors have occurred, the intracellular signal transduction of IL-6 is delivered to the Janus Kinase 2 (Jak2) tyrosine kinase and the Signal Transducers and Activators of Transcription 3 (Stat3) [12]. Signals from the gp130, Jak2, and Stat3 core components have numerous impacts on cellular activities in a context-dependent manner, such as inducing crosstalk with the Toll-Like Receptor (TLR), Mitogen-Activated Protein Kinase (MAPK), and NF-κB, thus causing a pro-inflammatory commitment [12,18,19,20,21]. Conversely, IL-6 can also coordinate anti-inflammatory activities through the Suppressors of Cytokine Signaling (SOCS) as a negative regulator of Jak2 and Stat3 [12]. Overall, the context-dependent and complex properties of IL-6 underscore the necessity to gain a more thorough understanding of its mechanisms of action prior to exploring its potential translational roles in disease-oriented application.

In stroke patients, serum IL-6 levels are increased and positively correlated with clinical outcome [22,23]. In rodent studies, cerebral ischemia causes an elevated expression of IL-6 in the injured brain and blood concentration. Furthermore, either with the reduction of IL-6 levels through anti-inflammatory treatments or the intravenous injection of IL-6 neutralizing antibodies, cerebral ischemic damage can be alleviated [24,25,26,27]. Besides this, the inhibition of the Jak2/Stat3 pathway that can be activated with excess IL-6 during the acute phase of cerebral ischemia confers neuroprotection in ischemic stroke [28,29,30,31,32,33]. Taken altogether, these findings indicated the deleterious effects of IL-6 on neuronal injury after cerebral ischemia. However, some other studies also show that the early intravenous or intracerebroventricular injection of recombinant IL-6 can improve cerebral ischemia [34,35,36], and the IL-6 signaling component, gp130, may mediate neuroprotective and anti-inflammatory effects against cerebral ischemia [37,38]. In addition to involvement in neuronal damages in acute stroke, IL-6 has been reported to induce skeletal muscle inflammation-associated insulin resistance and glucose intolerance in obesity [15,16,39]. However, whether IL-6 contributed to metabolic abnormalities after acute stroke was less clear.

At our laboratories, we had published serial studies showing that through anti-inflammation strategies, either directly (e.g., by Tumor Necrosis Factor-α receptor (TNFR) antagonist) or indirectly (e.g., by propranolol), central neuronal injuries, hyperglycemia, insulin resistance, as well as inflammation in the brain, liver, and skeletal muscles can be reduced after acute stroke in a cerebral ischemia rodent model [40,41,42,43]. Accordingly, based on the multiple pathophysiological effects of IL-6, we hypothesized that increased IL-6 expression after acute stroke may be involved in the poststroke inflammation of multiple organs such as the brain, liver, and skeletal muscles, as well as in metabolic abnormalities. This study centered on the IL-6 signaling Jak2/Stat3 pathway, in consideration of its important role for the regulation of immune responses and involvement in many pathological processes. We used a Jak2 inhibitor, AG490, to explore its effects on inflammation in the cerebral cortical, hepatic, and skeletal muscle tissues, and in the changes of the insulin signaling in a rat model of acute cerebral ischemia. The results of this study may be helpful for a better understanding of the pathogenic role of IL-6 in inflammatory and metabolic disorders after acute stroke.

## 2. Materials and Methods

### 2.1. Animal Allocation and Cerebral Ischemia

The Animal Experimental Committee of Taichung Veterans General Hospital reviewed and approved all animal protocols (IACUC approval code: La-1071584; IACUC approval date: 1 August 2018). Adult male Sprague–Dawley rats (10 weeks old and weighing 300–330 g), purchased from BioLASCO (Taipei, Taiwan), were allocated to four groups: Sham/Saline (*n* = 32); Ischemia/Saline (*n* = 32); Sham/AG490 (*n* = 32); and Ischemia/AG490 (*n* = 32). Under anesthesia with isoflurane (2–4%), the two common carotid arteries and the right middle cerebral artery of the rats were occluded to produce permanent cerebral ischemia using the method described in our previous study [40]. The sham groups were treated with all surgical procedures except for the arterial occlusion. Ischemia and sham groups received an intraperitoneal injection of normal saline or AG490 (5 mg/kg) 30 min prior to surgery. AG490 was administered according to the protocol and dosage described in a previous study of cerebral ischemia reperfusion injury in rats and evaluated in pilot tests [34,36]. The duration of ischemia was 24 h and all rats were euthanized for analyses. To further demonstrate the altered tissue expression of IL-6 in cerebral ischemia rats, β-adrenergic receptor antagonist propranolol (2 mg/kg) and TNF-α receptor inhibitor R-7050 (5 mg/kg) were delivered as per the same protocol of AG490. Under anesthesia with isoflurane (4%), rats were euthanized and decapitated according to our previous reports [40,43]. The obtained brain cortexes, livers, and gastrocnemius muscles were allocated to further analyses. The blood was withdrawn from the left femoral artery and frozen at −70 °C.

### 2.2. Neurological Evaluation

The neurological deficit was evaluated based on the sensorimotor performance (*n* = 8 per group) in accordance with our previous study [40]. The performance was evaluated by a blind evaluator using a modified six-point scoring criteria graded by neurological deficit.

### 2.3. Quantification of Ischemic Infarction

The dissected brains (*n* = 8 per group) were placed in a cooled Brain Slicer Matrix, and cut coronally at 2-mm intervals. The cut brain tissues were incubated in a 2% Triphenyltetrazolium Chloride (TTC) solution at 37 °C for 30 min to stain the viable tissues [40]. The infarct areas were delineated using Image J software (National Institutes of Health, Bethesda, MA, USA).

### 2.4. Brain Edema

The ipsilateral cortical tissues of ischemic brains (*n* = 8 per group) were weighed and then dried at 110 °C for 24 h in an oven. A wet/dry weight was measured to determine water content [40].

### 2.5. Measurement of Oxidative Stress

The malondialdehyde (MDA) levels of ipsilateral cortical tissues, gastrocnemius muscles, and livers (*n* = 8 per group) were measured with a Thiobarbituric Acid-Reactive Substance (TBARS) assay kit (Abcam, Cambridge, UK) according to the manufacturer’s instructions, and were used as an index for the lipid peroxidation product.

### 2.6. Caspase-3 Activity Assay

The caspase-3 activity in the ipsilateral cortical tissues was measured with a Fluorometric Assay Kit (BioVision, Mountain View, CA, USA) according to the manufacturer’s instructions.

### 2.7. Glucose Tolerance Test

After 8 h fasting, Intraperitoneal Glucose Tolerance Test (IPGTT) was performed through the administration of a glucose solution (2 g/kg) in the rats (*n* = 8 per group). A hand-held Accu-Check glucometer (Roche Diagnostics, Indianapolis, IN, USA) was used to measure the glucose levels over a 2 h period from the tail veins. The total Area Under Curve (AUC) of the glucose and time was calculated.

### 2.8. Blood Sample Analyses

The plasma insulin (Shibayagi, Gunma, Japan), C-Reactive Protein (CRP), and free fatty acids (R&D Systems, Minneapolis, MN, USA) levels (*n* = 8 per group) were measured with Enzyme-Linked Immunosorbent Assay (ELISA) kits according to the manufacturer’s instructions.

### 2.9. Measurement of Tissue Cytokines

The levels of Tumor Necrosis Factor-α (TNF-α), Interleukin-1β (IL-1β), and IL-6 in ipsilateral cortical tissues, gastrocnemius muscles, and livers (*n* = 8 per group) were measured with ELISA kits (R&D Systems, Minneapolis, MN, USA).

### 2.10. Western Blot Analysis

The ipsilateral cortical tissues, gastrocnemius muscles, liver tissues, and bEnd.3 cell lysates were homogenized using a Tissue Protein Extraction Reagent (Pierce Biotechnology, Rockford, IL, USA). Equal amounts of extracted proteins were separated through a standardized SDS-PAGE (8% and 12%) and transferred onto PVDF membranes, which were sequentially incubated with 5% skim milk, corresponding with primary antibodies, IgG-HRPs, and enhanced chemiluminescence Western blotting reagents (*n* = 8 per group). The chemiluminescence on the membranes were visualized using a G:BOX mini multi fluorescence and chemiluminescence imaging system (Syngene, Frederick, MD, USA) and quantified by Image J software (National Institute of Health, Bethesda, MD, USA). Primary antibodies were recognized, which included Receptor-Interacting Protein Kinase 1 (RIPK1, 1:1000), Microtubule-Associated Protein 2 (MAP-2, 1:1000), Cluster of Differentiation 68 (CD68, 1:1000), Glial Fibrillary Acidic Protein (GFAP, 1:1000), *Ubiquitin* Protein *Ligase E3* Component N-Recognin 1 (Ubr1, 1:1000), Tumor Necrosis Factor-α Receptor Type I (TNFRI, 1:1000), IKK-α/β (1:1000), p65 (1:1000), phospho-p65 (Serine-536, 1:500), Stat3 (1:1000), phospho-Stat3 (Tyrosine-705, 1:500), Zonula Occludens-1 (ZO-1, 1:1000), c-Jun N-terminal Kinase (JNK, 1:1000), phospho-JNK (Threonine-183/Tyrosine-185, 1:500), Akt (1:1000), phospho-Akt (Serine-473, 1:500), Janus Kinase 2 (Jak2, 1:1000), phospho-Jak2 (Tyrosine-1007, 1:1000), Insulin Receptor Substrate-1 (IRS1, 1:1000), phospho-IRS1 (Serine-307, 1;500), Suppressors of Cytokine Signaling 3 (SOCS3, 1:1000), Synaptosome Associated Protein 25 (SNAP25, 1:1000), Glyceraldehyde 3-Phosphate Dehydrogenase (GAPDH, 1:3000) (Santa Cruz Biotechnology, Santa Cruz, CA, USA), phospho-IKK-α/β (Serine-176/180, 1:500), and phospho-IRS1 (Tyrosine-895, 1:500) (Cell Signaling, Beverly, MA, USA).

### 2.11. Zymography Assay

Equal amounts of extracted proteins (the same as Western blot) from ipsilateral cortical tissues were separated through a standardized SDS-PAGE (8%) (*n* = 8 per group). The electrophoretic gels were washed with 2.5% Triton X-100, incubated in a buffer (25 mM Tris, 150 mM NaCl, 10 mM CaCl_2_, 0.2% Brij-35, pH 7.5), and stained with Coomassie brilliant blue R-250 (0.2%). The intensities of the visualized bands were quantified by Image J software (National Institutes of Health, Bethesda, MA, USA).

### 2.12. Cell Cultures

The immortalized mouse brain bEnd.3 endothelial cells purchased from the Bioresource Collection and Research Center (BCRC number: 60515, Hsinchu, Taiwan) were maintained in Dulbecco’s Modified Eagle Medium (DMEM) with 10% Fetal Bovine Serum (FBS) at 37 °C and 5% CO_2_. Cells were treated with a vehicle of recombinant IL-6 (50 ng/mL), AG490 (50 µM), MG132 (5 µM), or in combination, for 24 h.

### 2.13. Measurement of Endothelial Barrier Integrity

Transendothelial Electrical Resistance (TEER) and transendothelial permeability to dextran-FITC were measured in a Transwell apparatus [43,44]. The bEnd.3 cells were seeded onto Transwell inserts and grown to confluence. The TEER of the cell monolayer was measured with a Millicell ERS ohmmeter (Millipore, Billerica, MA, USA). The upper chambers were loaded by dextran-FITC (0.1 µg/mL) for 30 min, and its content in the lower chambers was measured using a fluorometer (Ex 492 nm and Em 520 nm).

### 2.14. Statistical Analysis

All the data were expressed as Mean ± Standard Deviation. A two-way analysis of variance, followed by Dunnett’s or Tukey post-hoc test, was performed for a group comparison using GraphPad Prism software (San Diego, CA, USA). A *p* value less than 0.05 was considered statistically significant.

## 3. Results

### 3.1. AG490 Alleviated Poststroke Brain Injury

**Figure 1:**
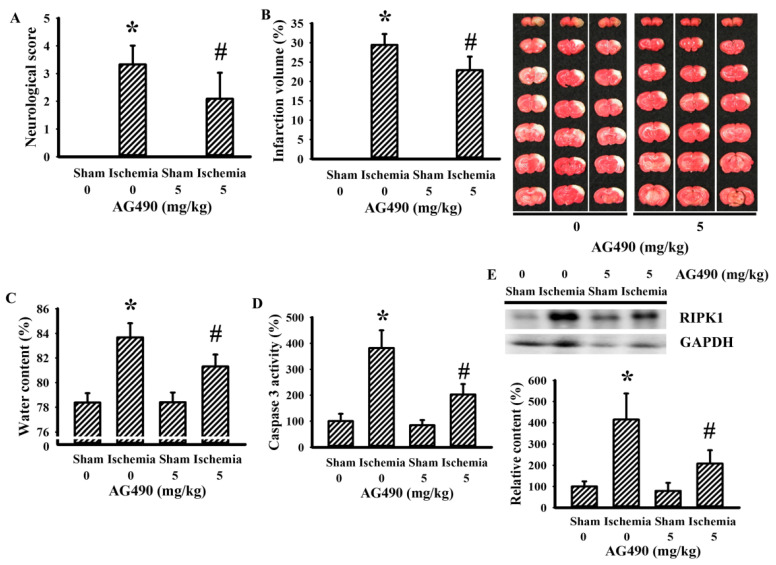
AG490 improved cerebral ischemia injury: (**A**) neurological deficits after permanent cerebral ischemia for 24 h in sham and stroke rats receiving normal saline or AG490 (5 mg/kg); (**B**) average infarction volume of ipsilateral hemisphere by TTC staining; (**C**) water contents in the ipsilateral cortical tissues; (**D**) caspase-3 activity by an enzymatic assay in the ipsilateral cortical tissues; and (**E**) Western blot of the ipsilateral cortical tissue proteins with the indicated antibodies and the quantitative results. * *p* < 0.05 vs. sham/saline; ^#^ *p* < 0.05 vs. ischemia/saline, *n* = 8.

The Jak2 inhibitor AG490 has been used in investigations of cerebral ischemia to explore the role of IL-6 [34,36,45]. In this study, the blockade of potential IL-6/Jak2 signaling was produced through the intraperitoneal introduction of AG490 30 min prior to cerebral ischemia. As with previous studies [34,36,45], the impaired sensorimotor performance (Figure 1A) and ipsilateral development of brain infarction (Figure 1B), brain edema (Figure 1C), and caspase-3 activation (Figure 1D) in rats with cerebral ischemia were alleviated by AG490. Elevated expression of necroptotic RIPK1 has been implicated in cerebral ischemia brain injury [46]. Its elevated expression was alleviated by AG490 (Figure 1E). The findings indicate a neuroprotective effect of AG490 pretreatment against ischemic brain injury.

### 3.2. AG490 Alleviated Poststroke Oxidative Stress and Inflammation

**Figure 2:**
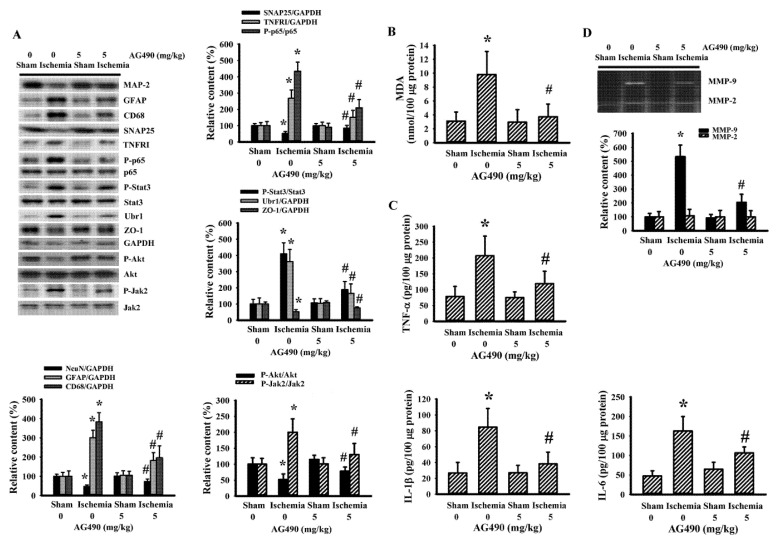
AG490 diminished poststroke brain oxidative stress and inflammation: (**A**) Western blot of the ipsilateral cortical tissue proteins with the indicated antibodies and the quantitative results in sham and stroke rats receiving normal saline or AG490 (5 mg/kg); (**B**,**C**) MDA contents, and TNF-α, IL-1β, and IL-6 protein levels in the ipsilateral cortical tissues; And (**D**) zymography assay of MMP-2 and MMP-9 activity of the ipsilateral cortical tissue proteins and the quantitative results. * *p* < 0.05 vs. sham/saline ^#^ *p* < 0.05 vs. ischemia/saline, *n* = 8.

Since oxidative stress and inflammation have substantial roles in the expansion of poststroke brain injury [7,8], changes in the accompanying biochemical events were investigated in cortical tissues ipsilateral to cerebral ischemia with the aim of further exploring the neuroprotective effects of AG490. The injured cortical tissues of rats with cerebral ischemia exhibited decreased protein expression of neuron-related MAP-2 and SNAP25, though there was evidence of the increased protein expression of macrophage/microglia-related CD68 and astrocyte-related GFAP (Figure 2A). Concurrent alterations were found with an elevation of MDA (Figure 2B), and increases of TNFRI protein expression, NF-κB p65 protein phosphorylation, Jak2 protein phosphorylation, and Stat3 protein phosphorylation (Figure 2A), as well as tissue TNF-α, IL-1β, and IL-6 protein expression (Figure 2C). Conversely, there was a reduction of Akt protein phosphorylation and tight junction ZO-1 protein expression (Figure 2A). The reduction of ZO-1 was paralleled by increased protein expression in ubiquitin Ubr1 E3 ligase (Figure 2A), along with enhanced MMP-9 activity (Figure 2D). The changes in ipsilateral cortical tissues were reversed by AG490 (Figure 2). Our findings suggested that AG490 induced a reversal effect on poststroke neural cell alteration, oxidative stress, inflammation, and Blood–Brain Barrier (BBB) disruption.

### 3.3. AG490 Improved Poststroke Hyperglycemia

**Figure 3:**
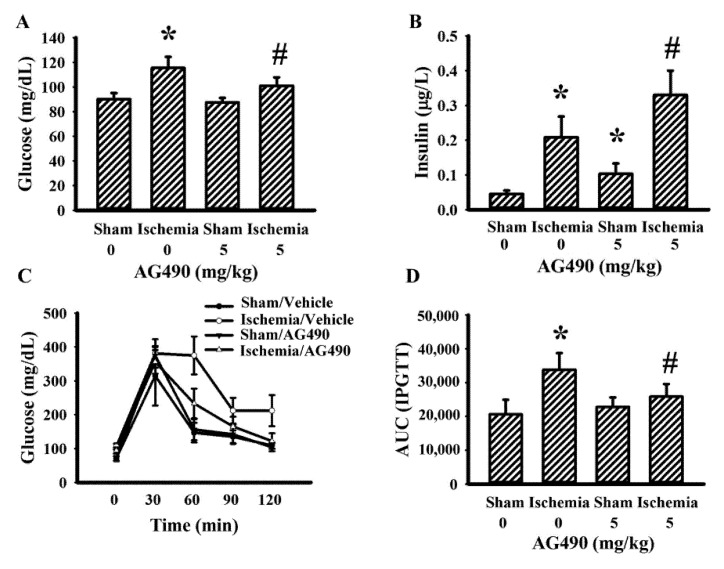
AG490 alleviated poststroke hyperglycemia: (**A**) blood glucose, (**B**) insulin after 8 h fasting in sham and stroke rats receiving normal saline or AG490 (5 mg/kg); (**C**) Blood glucose levels after intraperitoneal administration of glucose solution (2 g/kg); and (**D**) AUC of the glucose-time curves. * *p* < 0.05 vs. sham/saline; ^#^ *p* < 0.05 vs. ischemia/saline, *n* = 8.

**Figure 4:**
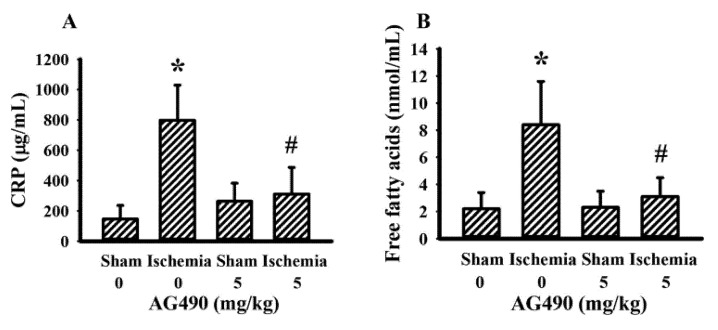
AG490 alleviated poststroke blood concentrations: (**A**) CRP, (**B**) free fatty acids in sham and stroke rats receiving normal saline or AG490 (5 mg/kg). * *p* < 0.05 vs. sham/saline; ^#^ *p* < 0.05 vs. ischemia/saline, *n* = 8.

The IL-6/Jak2 inflammatory axis has been implicated in the impairment of insulin signaling [12,14,39]. Therefore, the effects of AG490 on poststroke glucose metabolism were investigated. As shown in our previous reports [40,41,42,43], rats with cerebral ischemia developed hyperglycemia (Figure 3A), hyperinsulinemia (Figure 3B), and glucose intolerance (Figure 3C,D). AG490 displayed an alleviative effect on hyperglycemia (Figure 3A) and post-load glucose levels (Figure 3C,D), and augmented hyperinsulinemia (Figure 3B). In systemic parameters linked to glucose metabolism, cerebral ischemia resulted in increased circulation levels of CRP (Figure 4A) and free fatty acids (Figure 4B) in rats, and the increments were alleviated by AG490. These findings suggest AG490 confers a beneficial effect against poststroke hyperglycemia and impaired glucose tolerance.

### 3.4. Cerebral Ischemia Impaired Insulin Action in Gastrocnemius and Liver as Well as a Reversal Effect of AG490

**Figure 5:**
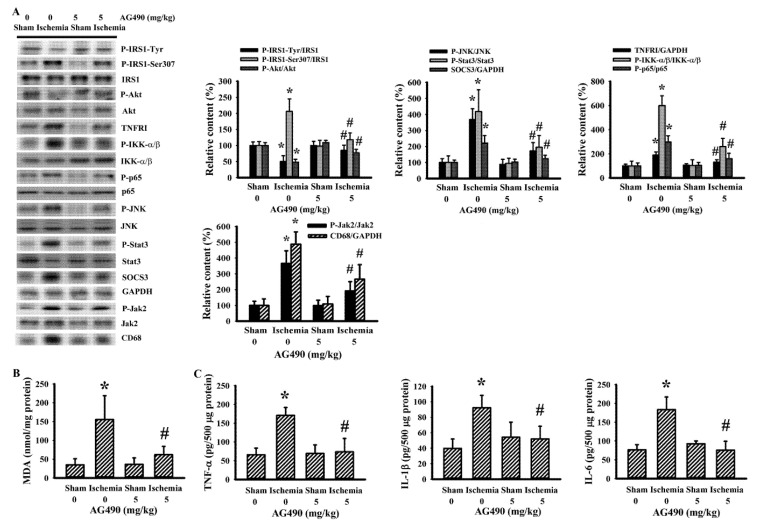
AG490 alleviated poststroke gastrocnemius inflammation: (**A**) Western blot of gastrocnemius muscle proteins with the indicated antibodies and the quantitative results in sham and stroke rats receiving normal saline or AG490 (5 mg/kg); (**B**,**C**) MDA contents, and TNF-α, IL-1β, and IL-6 protein levels in the gastrocnemius muscles. * *p* < 0.05 vs. sham/saline; ^#^ *p* < 0.05 vs. ischemia/saline, *n* = 8.

**Figure 6:**
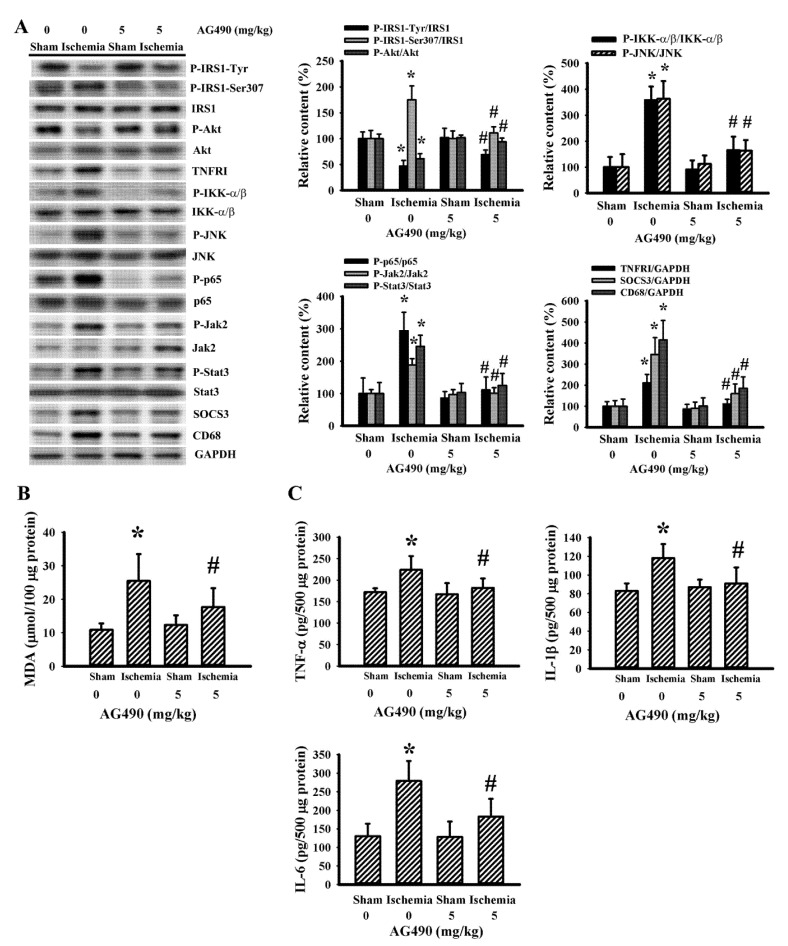
AG490 alleviated poststroke hepatic inflammation: (**A**) Western blot of liver proteins with the indicated antibodies and the quantitative results in sham and stroke rats receiving normal saline or AG490 (5 mg/kg); (**B**,**C**) MDA contents, and TNF-α, IL-1β, and IL-6 protein levels in the liver. * *p* < 0.05 vs. sham/saline; ^#^ *p* < 0.05 vs. ischemia/saline, *n* = 8.

Skeletal muscles are targets of peripheral insulin and are central to postprandial blood glucose uptake. IL-6/Jak2 signaling adversely interferes with the action of insulin, resulting in glucose intolerance and insulin resistance [12,15,16,47]. There was a reduction in active IRS1-associated tyrosine phosphorylation and Akt phosphorylation, and an increase in inhibitory IRS1-associated serine phosphorylation in the gastrocnemius muscles following cerebral ischemia, and the changes were alleviated by AG490 (Figure 5A). The decreased insulin signaling and reversal effects of AG490 were paralleled by alterations in Jak2 protein phosphorylation, Stat3 protein phosphorylation, SOCS3 protein expression, TNFRI protein expression, JNK protein phosphorylation, IKK-α/β protein phosphorylation, NF-κB p65 protein phosphorylation, CD68 protein expression (Figure 5A), MDA production (Figure 5B), and tissue protein expression in TNF-α, IL-1β, and IL-6 (Figure 5C). The altered parameters in the postischemic gastrocnemius muscles were alleviated by AG490 (Figure 5). The liver is also critical to stroke-associated dysmetabolism [42]. Cerebral ischemia impaired insulin action, and a reversal effect of AG490 was duplicated in the liver (Figure 6). Therefore, cerebral ischemia has an adverse effect on the insulin signaling in the gastrocnemius muscles and liver involving oxidative stress and inflammation, with AG490 improving the impairment.

### 3.5. Propranolol and R-7050 Alleviated Tissue IL-6 Expression

**Figure 7:**
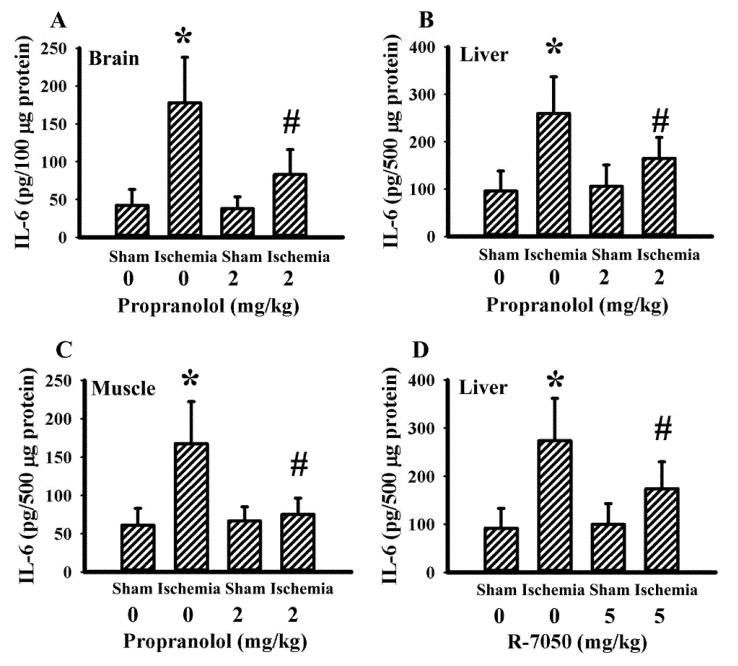
Propranolol and R-7050 alleviated tissue IL-6 expression in cerebral ischemia rats. IL-6 protein levels in: (**A**) brain cortex; (**B**) liver; and (**C**) gastrocnemius muscles in sham and stroke rats receiving normal saline or propranolol (2 mg/kg) intraperitoneal injection. * *p* < 0.05 vs. sham/saline; ^#^ *p* < 0.05 vs. ischemia/saline, *n* = 6. (**D**) Liver IL-6 protein levels in sham and stroke rats receiving normal saline vehicle or R-7050 (5 mg/kg) intraperitoneal injection. * *p* < 0.05 vs. sham/saline; ^#^ *p* < 0.05 vs. ischemia/saline, *n* = 8.

Our previous studies revealed that both propranolol and R-7050 protected rats against cerebral ischemia-induced metabolic and inflammatory changes as well as brain injuries. The elevation of IL-6 expression in brain cortical tissues and gastrocnemius tissues was alleviated by R-7050, while the effects of propranolol on tissue IL-6 expression remained undetermined [40,43]. Tissues in both previous studies revealed an elevated IL-6 expression in the brain cortex, liver, and gastrocnemius of cerebral ischemia rats. Propranolol had an alleviative effect on IL-6 expression in brain cortical tissues (Figure 7A), liver tissues (Figure 7B), and gastrocnemius tissues (Figure 7C). In addition to the brain cortex and gastrocnemius [40,43], R-7050 also caused a reduction in liver IL-6 expression (Figure 7D). Therefore, the reduction of tissue IL-6 expression appears to be common upon beneficial intervention after cerebral ischemia.

### 3.6. AG490 Alleviated IL-6-Induced Endothelial Barrier Disruption

**Figure 8:**
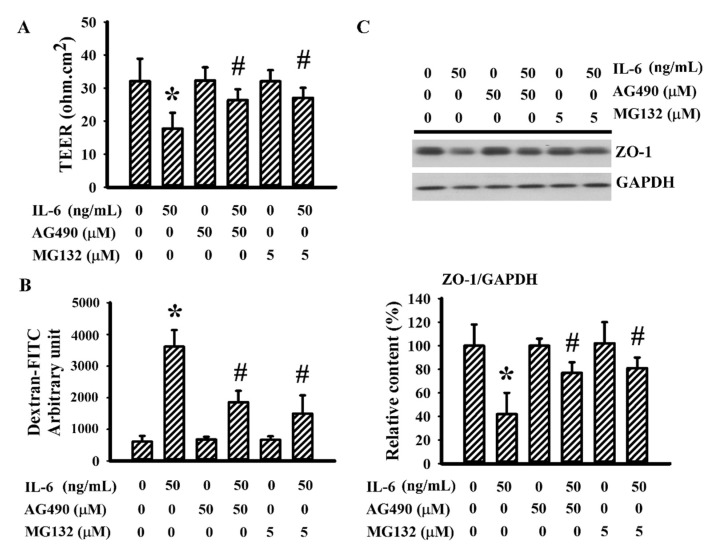
AG490 reduced endothelial permeability increased by IL-6 in bEnd.3 cells: (**A**) TEER, (**B**) permeability to dextran-FITC in bEnd.3 cells pretreated with vehicle, AG490 (50 µM), or MG132 (5 µM) for 30 min before incubation with recombinant IL-6 (0 and 50 ng/mL) for an additional 24 h; (**C**) Western blot of bEnd.3 extracted proteins with ZO-1 antibody and the quantitative results. * *p* < 0.05 vs. untreated control; ^#^ *p* < 0.05 vs. IL-6 control, *n* = 4.

IL-6 is a disruptor of endothelial barrier integrity [44,48]. Therefore, the potential contribution of AG490 on endothelial cell permeability was explored in a bEnd.3 endothelial cell model. Sustained IL-6 exposure caused endothelial barrier disruption, as evidenced by decreased TEER (Figure 8A), increased permeability to dextran-FITC (Figure 8B), and lowered ZO-1 protein (Figure 8C). The presence of AG490 alleviated endothelial barrier disruption caused by IL-6 (Figure 8). Moreover, the proteasome inhibitor MG132 [44] also displayed alleviative effects against IL-6-disrupted endothelial barrier integrity (Figure 8). The findings suggest that the prevention of endothelial dysfunction and BBB disruption by decreasing the reduction of tight junction ZO-1 protein is probably a neuroprotective mechanism of AG490.

## 4. Discussion

In our study, rats with cerebral ischemia exhibited increased IL-6 expression and Jak2 downstream Stat3 protein phosphorylation in the injured cortical tissues, livers, and gastrocnemius muscles. The introduction of the Jak2 inhibitor AG490 blocked IL-6/Jak2 signaling, resulting in the amelioration of the poststroke neurological deficit, brain infarction, brain edema, oxidative stress, pro-inflammatory cytokine expression, and caspase-3 activation. The neuroprotective effects of AG490 in the injured cortical tissues correlated well with a reduction of Stat3 signaling, TNFRI signaling, NF-κB signaling, ubiquitin Ubr1 E3 ligase signaling, and MMP-9 signaling, as well as an increased tight junction ZO-1 protein expression. Furthermore, the endothelial barrier protective effects of AG490 were demonstrated in in vitro IL-6-exposed bEnd.3 endothelial cells which showed a decrease in ZO-1 decline. Besides this, the administration of AG490 decreased fasting glucose, glucose tolerance impairment, plasma CRP, and plasma-free fatty acids, with a parallel improvement of insulin action in the liver and gastrocnemius muscles as well as reduced oxidative stress, Stat3 signaling, JNK signaling, NF-κB signaling, and inflammation. The overall findings provide experimental evidence of using Jak2 blockade therapy, which can modulate central and peripheral inflammatory responses and metabolic disorder after acute ischemic stroke.

The recruitment of Jak2 by IL-6R and the gp130 receptor complex, and communication with Stat3, are essential to the biological execution of IL-6 [12,16,19]. However, it should be noted that in addition to IL-6, other factors such as leukemia inhibitory factor, ciliary neurotrophic factor, growth hormones, and leptin can also modulate the Jak2/Stat3 signal transduction system [49]. Hence, the currently observed increased Jak-Stat3 phosphorylation after acute ischemia might not exclusively be restricted to stimulation by IL-6. Once cerebral ischemia occurs, dramatic changes in local cell metabolism create niches whereby numerous intracellular signaling cascades are triggered as an adaptive response. If the adaption is imbalanced, these signals may gradually lead to the death of neighboring parenchymal cells via apoptosis, autophagy, necroptosis, pyroptosis, or ferroptosis [46,50,51,52]. Among the surrogate candidates, the Jak2 signaling pathway is crucial in cerebral ischemia cell adaption and death. Cerebral ischemia causes activation of the Jak2 signaling pathway, which is closely related to the occurrence of poststroke brain injury. Inhibiting activation of the Jak2 signaling pathway can inhibit neuronal apoptosis, thereby alleviating brain injury [28,29,30,31,32]. Conversely, the activation of the Jak2 signaling pathway is also pivotal in the cerebral ischemia neuroprotective actions of melatonin, resveratrol, leptin, and erythropoietin derivatives [53,54,55,56]. In our rat model of permanent cerebral ischemia, pretreatment with the Jak2 inhibitor AG490 protected brains from poststroke apoptosis and injury, although the putative protective or deleterious roles of the Jak2 signaling pathway in cerebral ischemia have yet to be determined.

The IL-6/Jak2/Stat3 axis has also been implicated in endothelial permeability and poststroke BBB disruption involving the MMP- or ubiquitin proteasome-mediated degradation of tight junction proteins [25,44,48]. Rats with cerebral ischemia had decreased endothelial tight junction ZO-1 proteins, which was reversed by AG490 with increased ubiquitin Ubr1 E3 ligase and MMP-9 activity. In parallel, the endothelial barrier-protective effects of AG490 via the targeting of ZO-1 protein content were demonstrated in IL-6-stimulated bEnd.3 endothelial cells. Although antibody neutralization and exogenous addition studies were not conducted, the combined in vitro and in vivo findings confirmed the blockade of Jak2 inflammatory signaling and consequences through AG490, and implied a potential involvement of the IL-6/Jak2/Stat3 axis. 

Transcription factor Stat3 is a critical target of the Jak2 signaling pathway which operates through tyrosine phosphorylation-mediated activation, and is central to cellular adaption. Stat3 has been shown to be activated in in vitro and in vivo experimental models of stroke and promotes transcriptional upregulation of numerous genes that may play a critical role in both neural injury and repair [32,57]. The conflicting findings complicate the specific role that Stat3 plays in the pathogenic processes of cerebral ischemia. Accumulating evidence indicates that the aberrant-activated Stat3 can promote the transcription and expression of pro-inflammatory mediators, including cytokines, chemokines, and adhesion molecules through direct promoter targeting or via cooperation with an epigenetic modifier such as Jumonji Domain-Containing Protein D3 (*JMJD3*) [24,58]. Additionally, its mutual interaction with the TLR4 signaling pathway represents an alternative mechanism of Stat3 that drives inflammatory responses and oxidative stress [19]. Conversely, M2 macrophage polarization and the protein expressions of SOCSs and TNF-α-induced Protein 3 (TNFAIP3 or A20) deubiquitinase contribute to the anti-inflammatory effects of the Stat3 signaling pathway [21,28,59,60,61].

After cerebral ischemic damage, an increasing number of endogenous host-derived molecules, termed damage-associated molecular patterns, and cytokines in the brain leak into the circulation and trigger systemic immunity and inflammation in multiple peripheral organs, such as bone marrow, the spleen, lymph nodes, and gut [62]. Additionally, this study showed that inflammatory response also occurred in the liver and skeletal muscles in association with an enhanced phosphorylation of Jak2/Stat3 and parallel increases in TNFRI, NF-κB signaling, oxidative stress, and the cytokine proteins of TNF-α, IL-1β, and IL-6 soon after ischemic brain injury, in line with our previous report [42,43]. It is proposed that infiltrated leukocytes or activated resident macrophages in peripheral tissues may be one of the sources of peripheral proinflammatory cytokine production after brain injury [62]. On the contrary, these hepatic and muscular inflammatory changes were alleviated by AG490. However, whether decreased peripheral inflammation was medicated by the direct effects of AG490 on the target organs, or indirectly by less cerebral neuronal injury, requires further clarification.

Glucose intolerance and insulin resistance are closely linked to low-grade chronic inflammation and higher circulating levels of free fatty acids [15,43]. Besides this, regarding the inflammation-associated impairment of glucose metabolism, SOCSs, IKK-α/β, and JNK are plausible mechanistic links. Upon insulin binding, the Insulin Receptor (IR) is phosphorylated at the tyrosine residue, which then causes tyrosine phosphorylation in IRS1 leading to Akt activation, a process central to glucose transporter membrane shuttling and glucose metabolism. SOCSs antagonize the activation of IR and IRS1, while IKK-α/β and JNK interfere with the activation of IRS1 by adding a phosphate group to the inhibitory Serine-307 (rodent)/Serine-312 (human) moiety in the liver and skeletal muscles, both of which have fundamental roles in postprandial glucose uptake and storage through conventional insulin actions [15,42,63]. In our study, AG490-mediated improvement in fasting blood glucose and glucose intolerance was accompanied by reduction of the plasma levels of CRP and free fatty acids, as well as an increased circulation of insulin after acute stroke, which implies an anti-inflammatory mechanism attempts to resolve the impairment of glucose metabolism. Furthermore, the metabolic improvement effects of AG490 were evident in the signaling pathway in the liver and gastrocnemius muscles of stroke rats. In stroke rats, it was shown that IRS1 tyrosine phosphorylation and Akt phosphorylation were reduced along with increased IRS1 Serine-307 phosphorylation, Jak2 phosphorylation, Stat3 phosphorylation, SOCS3, TNFRI, IKK-α/β phosphorylation, JNK phosphorylation, oxidative stress, and protein expression in TNF-α, IL-1β, and IL-6 in the liver, and gastrocnemius muscles. In contrast, these alterations were alleviated by AG490. Fbxo40, a muscle-specific E3 ubiquitin ligase targeting the IRS1 for degradation, is activated by Stat3 and contributes to muscular insulin resistance [47]. We speculated that there was relatively little involvement of Fbxo40 in cerebral ischemia-associated hepatic and muscular insulin resistance because IRS1 content remained constant among the groups. Overall, the positive effects of AG490 in terms of improving poststroke hyperglycemia and glucose intolerance could be attributed to its inhibitory effects on hepatic and muscular inflammation.

Regarding the inflammatory properties of cytokines, it should be noted that IL-6 has two opposing effects on glucose metabolism. Because plasma IL-6 level is higher in subjects with obesity, metabolic disease, or insulin resistance, it is generally accepted that depletion of IL-6 improves glucose regulation [13,64]. However, plasma IL-6 levels are rapidly elevated during exercise, and exercise is an effective way to alleviate insulin resistance [65]. Studies have reported that short-term treatment with IL-6 improves insulin-induced glucose uptake in skeletal muscles, although sustained treatment with IL-6 causes glucose intolerance and insulin resistance [15,39]. Our findings revealed that poststroke hyperglycemia, glucose intolerance, and brain injury were linked to high IL-6 content, and the Jak2 inhibitor AG490 reversed these alterations. Nevertheless, additional targets of IL-6 signaling components, such as IL-6R and gp130, as well as Jak2/Stat3-associated anti-inflammatory and metabolic resolving effects, remain to be investigated. Thus, a deeper investigation highlighting the aforementioned phenomena is warranted.

There were some limitations in this study. During cerebral ischemia it has been reported that neuronal intracellular calcium levels are increased, and thus overload mitochondria with an increased production of reactive oxygen species, and oxidative stress and the necrotic death of brain cells. A recent study shows that an antioxidant enzyme, peroxiredoxin-6 (Prx-6), can protect oxygen–glucose deprived neuronal cells by reducing the expression of factors involved in apoptosis activation, such as caspase-3, and pro-inflammatory cytokines. Particularly, it also inhibits intracellular calcium increases, and thus alleviates neuronal injury [66]. Besides this, there are increasing amounts of studies describing the role of anti-inflammatory regulators for ischemic stroke. Interleukin-10 (IL-10), an anti-inflammatory cytokine, has been shown to be beneficial for neurogenesis in the ischemic brain by attenuating pro-inflammatory signals and upregulating anti-apoptotic proteins [67]. The transforming growth factor-β (TGF-β), another neuroprotective and anti-inflammatory mediator produced by astrocytes and microglia, when overexpressed, reduced ischemic brain injury in experimental studies, while brain damage was exacerbated when TGF-β was blocked [68]. The IL-1 receptor antagonist (IL-1Ra), a competitive antagonist of IL-1, was found to decrease the concentration of IL-6 and downstream the products of IL-1, and thus confer neuroprotection [68]. However, our study was limited in that changes of intracellular calcium after cerebral ischemia as well as the expression of IL-10, and the other anti-inflammatory factors were not examined. Whether the neuroprotective effects of the Jak2 blockade by AG490 also derives from the regulation of calcium signaling and anti-inflammatory cytokines needs further investigation. Clinically, human studies showed that in patients with rheumatoid arthritis who received the interleukin-6 receptor antagonist, the risk of myocardial infarction and stroke can be decreased [69]. Besides this, use of the Jak Kinase or IL-6 inhibitor can be associated with a reduction of carotid intimal thickness, and an improvement of microvascular endothelial functions [70]. These clinical studies demonstrated somewhat the effects of anti-inflammatory cytokines therapy on cerebrovascular diseases. However, despite our experimental study showing the beneficial effects of the IL-6 pathway blockade on acute stroke, to be more closely related to clinical practice, a design with an IL-6-blockade with different administration time points (e.g., after acute insults) is necessary to elucidate its effect on stroke-induced inflammatory and metabolic responses.

## 5. Conclusions

**Figure 9:**
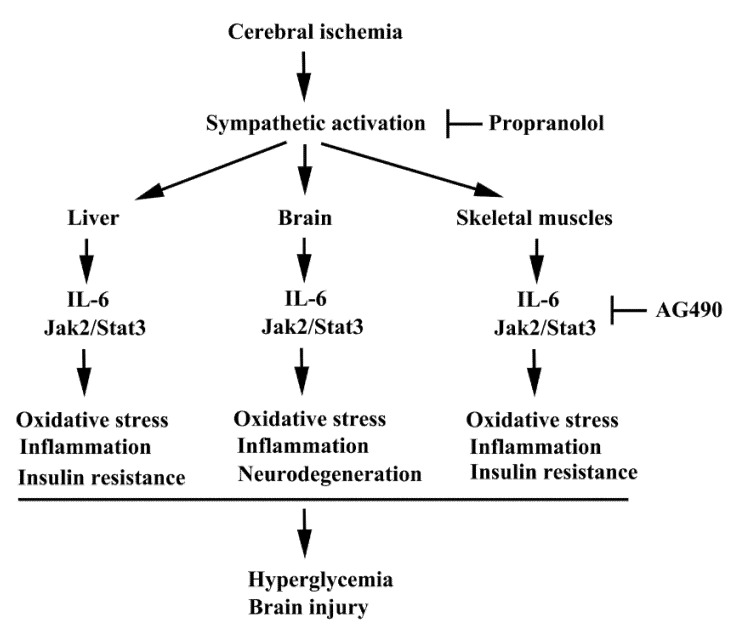
A possible schema of the molecular processes of poststroke inflammation and metabolic abnormality is proposed. In this study, the consequences of Jak2 intervention by AG490 in stroke rats are elicited. Beneficial effects of β-adrenergic receptor blockade by propranolol on poststroke inflammation and metabolic abnormality demonstrated by our previous study are also highlighted.

Hyperglycemia and inflammation are commonly linked to the expansion of poststroke brain injury. The pro-inflammatory cytokine, IL-6, is a plausible mechanistic link between chronic inflammation and glucose intolerance/insulin resistance via the IL6/Jak2/Stat3 axis. In this study, we provide experimental evidence in a rat cerebral ischemia model of the suppressive effects of the Jak2 inhibitor AG490 on brain injury, apoptosis, oxidative stress, neuroinflammation, hyperglycemia, glucose intolerance, skeletal/hepatic oxidative stress, skeletal/hepatic inflammation, and insulin resistance (Figure 9). Our findings further strengthen the concept that agents or strategies targeting inflammation, hyperglycemia, or both, may have a promising effect in preventing disease progression in conditions such as cerebral ischemia. These findings are encouraging, but further research is warranted to elucidate the precise mechanisms of AG490 and to identify additional targets beyond IL-6.

## Data Availability

Data is contained within the article.
